# Automatic Marker-free Longitudinal Infrared Image Registration by Shape Context Based Matching and Competitive Winner-guided Optimal Corresponding

**DOI:** 10.1038/srep39834

**Published:** 2017-02-01

**Authors:** Chia-Yen Lee, Hao-Jen Wang, Jhih-Hao Lai, Yeun-Chung Chang, Chiun-Sheng Huang

**Affiliations:** 1 Department of Electrical Engineering, National United University, Taiwan; 2 Institute of Biomedical Engineering, National Taiwan University, Taiwan; 3 Department of Medical Imaging, National Taiwan University Hospital and National Taiwan University College of Medicine, Taiwan; 4 Department of Surgery, National Taiwan University Hospital and National Taiwan University College of Medicine, Taiwan

## Abstract

Long-term comparisons of infrared image can facilitate the assessment of breast cancer tissue growth and early tumor detection, in which longitudinal infrared image registration is a necessary step. However, it is hard to keep markers attached on a body surface for weeks, and rather difficult to detect anatomic fiducial markers and match them in the infrared image during registration process. The proposed study, automatic longitudinal infrared registration algorithm, develops an automatic vascular intersection detection method and establishes feature descriptors by shape context to achieve robust matching, as well as to obtain control points for the deformation model. In addition, competitive winner-guided mechanism is developed for optimal corresponding. The proposed algorithm is evaluated in two ways. Results show that the algorithm can quickly lead to accurate image registration and that the effectiveness is superior to manual registration with a mean error being 0.91 pixels. These findings demonstrate that the proposed registration algorithm is reasonably accurate and provide a novel method of extracting a greater amount of useful data from infrared images.

The infra-red (IR) image has recently been considered as a potential assessment tool for chemotherapy evaluation and early cancer detection. Head *et al*. discussed the past, present, and future applications of IR imaging in medicine, including breast cancer^1^. Gautherie was the first to show that breast cancer patients with abnormal asymmetric breast infrared images had tumors with faster growth rates^2^. Arora *et al*. had shown that a modernized IR system can be a useful adjunctive tool in detecting breast cancer with high sensitivity by doing a prospective, double-blinded trial of 92 patients^3^.

Like fingerprints, heat patterns of each subject in the infrared image are distinctive. Observation of heat pattern changes on IR breast image during a long period can consequently assess the growth of breast cancer cells due to the higher temperature signature in comparison to their surrounding normal tissues, which are arising from the higher metabolic rate of cancerous tissue and its angiogenesis phenomena. Gautherie *et al*. did a long-term assessment of breast cancer risk in patients with a persistent abnormal thermogram via IR imaging. The examination results revealed that a persistent abnormal thermogram indicated a higher risk of cancer^4^. Sequential images with temporal information, i.e., longitudinal images, can reduce individual variation and characterize more accurately anatomic and functional changes in tumor growth to respond to chemotherapy monitoring or breast cancer detection by computing the IR energy of heat patterns.

Longitudinal IR image registration is an essential step toward the quantitative pixel-wise analysis of the heat energy and pattern change in a time course study. In longitudinal medical image registration, structural change of tumors or blood vessels can be seen on Magnetic Resonance Imaging (MRI) scans, which is why the over the past decade, topics of longitudinal medical image registration have mostly been associated with MRI brain scanning. For example, Csapo *et al*. proposed a model-based image similarity measure for longitudinal image registration in the presence of spatially non-uniform intensity change for MRI brain registration^5^. Holland *et al*. proposed a method to accurately quantify structural changes in organs based on serial MRI scans^6^. Because longitudinal images can reduce individual variation and detect more accurately functional or structural changes in sequential images with temporal information, it has been increasingly applied and focused clinically^7^.

Two general algorithms are available to image registration, namely rigid registration and non-rigid registration. Most medical images, such as this study, belong to non-rigid registration because the human body is soft and is highly deformed. Considerable research has been carried out previously for the development of various image registration methods. The general one of which is based on external conditions, which means that features are artificial and adhesive to target surfaces, such as markers, and that the registration can be performed without a complicated algorithm^8^,^9^. However, the dependence on external markers is not applicable to the registration of longitudinal medical images. Other registration methods regard the anatomic information of body surface as features. For medical images, enhancing image contrast can reinforce the robustness of the surface information and detect features and match those for registration. For example, the Head and Hat algorithm, surface matching technology, proposed by Pelizzari^10^ and the Iterative Closest Point algorithm^11^,^12^ directly extract surface features for registration as well. There are information-based image registration methods, such as the Fourier transform-based image registration technology proposed by Xu and Varshney^13^, where multiple signal classification with complicated calculation is used and a more accurate result could be obtained. Relevant technologies have been applied to CT image registration of brain and abdomen images^14^. Sanjay-Gopal *et al*.^15^ compared coefficients related to breast X-ray image registration and mutual information. To sum up, surface information-based image registration has become widely used to compare target changes at longitudinal research and is applied in this study.

The procedure of image registration in this study consists of the following steps: (1) feature detection (2) feature descriptor establishment and matching (3) optimal corresponding and transformation. Feature detection is hard to develop in IR images because of its properties of low contrast and weak edge. An appropriate feature detector is used to detect feature points in this paper. Since infrared breast images can reveal high temperature areas, i.e., heat pattern, which can be extracted vascular structure and regarded these structural intersection points as features. However, the vascular intersection is selected manually in the previous study^16^. In addition, modified Harris corner detector^17^ is adopted in this paper to extract feature points of infrared images, which is a detector with highly tolerant to rotation, zooming, illumination variation and anti-noise^18^, so it is reliable for IR images.

Feature point matching is a rather important step for image registration. Pairs of control points are established by feature descriptors. Therefore, describing the characteristics of feature points is essential for feature point matching. According to the description style, it can be roughly divided into spatial-frequency descriptor, differential descriptor, feature descriptor and others^19^. The spatial- frequency descriptor transfers images to frequency domain and captures such information as frequency and phase to form feature information including the Fourier descriptor^20^ and the wavelet descriptor^21^. The differential descriptor is established on differential calculations, such as differential invariant^22^, controllable filter^23^ and partial gray scale invariant^24^. The feature descriptor works with such surface characteristics as trend, intensity and shapes of image, including SIFT (scale invariant feature transform)^25^,^26^, PCA-SIFT^27^, GLOH (gradient location and orientation histogram)^28^, and Shape Context^29^,^30^. Proved by comparison to be superior to other descriptors, feature descriptor is most important and widely-used^31^. In 1999, David Lowe proposed the famous SIFT. SIFT descriptor generates 128-dimension feature description, according to the gradient distribution around the feature points, and can record the trend features of gray scale in target areas. However, its excessively high descriptive dimension is likely to cause problems in implementation and effectiveness. SIFT-based improvement approaches have been proposed, such as PCA-SIFT, which uses principal component analysis to reduce the dimension of SIFT descriptor and GLOH algorithm, which uses the SIFT descriptor to transform polar coordinates to enhance distinctiveness. The above two algorithms are also insufficiently distinctive to be descriptors and excessively complex to calculate^32^. However, the feature descriptor also describes image shape features. Shape Context records the contour points at different distances and angles, which consequently describes the object contour reliably and effectively. Previous methods based on Shape Context were reviewed in^33^. Belongie *et al*.^34^ proposed to regard the method as a basic method to measure similarity between shapes and exploit it for object recognition, since such description on was offering a globally discriminative characterization. Kang *et al*.^35^ applied Shape Context to match the missing or damaged regions of an image, which figures out the original image shape. Mori and Malik^36^ stored the two-dimension images of human body obtained at different angles and used those to estimate the body pose by Shape Context matching as well as the movement chain variation model. In this way, they located joints of human body to evaluate and find out the structure and posture of human body and its position in the three-dimension space. The above illustration shows the strong effect and great performance of Shape Context descriptor for matching. Besides Shape Context matching, there is another proposed matching technique called open curve matching. Batler *et al*.^37^ used the generated corresponding point sequence and took a curve as the matching method to register two-dimension images. Ayache *et al*.^38^ proposed a matching approach for 3D medical images by calculating the targeted crest line that corresponds to anatomy features. This algorithm is relatively stable in rigid images, but as far as breast infrared images in this study are concerned, the curve matching is not applicable because of the softness and deformation in anatomy.

In terms of image transformation, Elsen *et al*. summarized some common models including rigid transformation, affine transformation, projective transformation and curved transformation^39^. Rigid transformation is utilized for relatively stable projects, like a house. Affine transformation as well as many factors like translation, rotation, scale are considered and suitable for stable- linear project. Projective transformation is almost exclusively used to register projection 2D image to 3D images. Curved transformation may be used when one of the images has to be deformed to fit the other image. Since the project of this study is human breast, which is non-linearity and unstable. Accordingly, this study adopts the non-linear thin plate spline (TPS)^40^,^41^ which belongs to the curved transformation model.

While many studies have worked on image registration, few studies have yet to overcome the problem of finding anatomic fiducial markers on a body surface in the infrared image and the issue of developing a robust longitudinal registration without markers for IR images. The literatures show that the heat pattern of each individual breast is unique in infrared images and it’s reasonable to adopt feature descriptor for description in this study. Gradient direction is taken as the descriptor for initial relaxation matching in order to quickly and effectively find out the most matched points. However, such a descriptor has been simplified and thus causes mismatched points easily. Therefore, this study employs Shape Context for a strict matching of feature points. The few and correct matched point pairs can be obtained and used to debug the previous relaxation matching. In addition, specific locations of pairs of control points via matching process affect the accuracy of registration. Competitive winner-guided optimal corresponding method is developed for that in this study.

Main aims of the paper:

- Development of an automatic feature detection algorithm, i.e., automatic vascular intersection detection, on breast surface without adhering label points (markers) to provide not only an alternative way for physicians, but also a more comfortable and convenient check-up way for subjects.
- Development of the automatic matching of feature points to be corresponding point pair algorithm instead of using manual matching to reduce the inspection analysis time and errors.
- Development of a new generation non-rigid image registration algorithm to verify its reliability and to make longitudinal registration faster and more accurate.
- The proposed algorithm is supposed to be applied to a sequence of IR images taken before and during chemotherapy courses, while also requiring that all IR images taken during chemotherapy can be reasonably registered. The registered IR images are computed to quantitatively analyze the chemotherapy responses in the future.

## Result and Discussion

Feature detection methods in this paper are consisted of the modified Harris corner detection algorithm which is developed in the previous research^17^, and the automatic vascular intersection detection algorithm. These two representative feature points in infrared breast can be used for feature point matching.

### Results of feature points detection

#### Results of modified Harris corner detection

**Figure 1:**
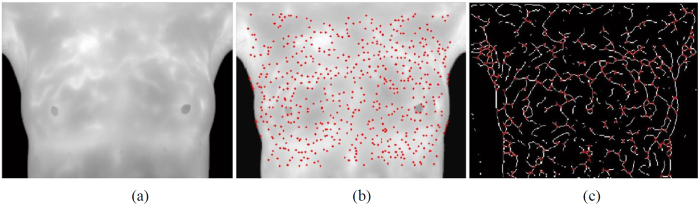
Results of detecting feature points in the infrared breast images, (**a**) Original image; (**b**) Results of the modified Harris Corner detection; (**c**) Results of the automatic vascular intersection detection.

Figure 1(a) shows the original image. Figure 1(b) shows the corner points (red points) detected by the modified Harris corner detector. After pretreating, the algorithm clearly detects an adequate number of corner points where located at bright areas of breast infrared image. Moreover, the addition of adaptive non-maximal suppression radius algorithm eliminates the less representative feature points in radius areas and solves the clustering problem of Harris corner points.

#### Results of automatic vascular intersection detection

To enhance the features of high-temperature tissues (areas featured as high level of gray scale in infrared images), this study thinned the high-temperature tissue areas to gain the vascular structure and strengthened it with the obtained vascular intersections. Figure 1(c) shows the results of the automatic detection of vascular intersections in infrared breast images. The white lines are vascular skeletons while the red points are vascular intersections. Naked-eye observation shows that all vascular skeleton intersections can be automatically found.

### Results of feature point matching

#### Results of relaxation matching based on gradient direction descriptor

**Figure 2:**
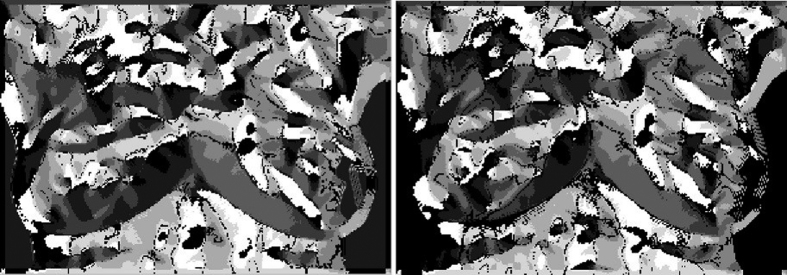
Gradient direction of infrared breast image.

Most feature points matching can be achieved in the matching process, which allows the existence of mismatching points is called relaxation matching, otherwise it is called strict matching. For relaxation matching of feature points, gradient direction descriptor is adopted in this study. Gradient direction descriptor calculates the gradient direction of each pixel in the window and records its gradient angle. Gradient direction is used as the template because there are similar gradient directions at different time points of IR images of human body, shown in Fig. 2. Template matching is used to examine where is there are the most similarities between the target image and the source image in the template. Usually, a part of the target image *T* is taken as the template *T*(*x, y*), where (*x, y*) is the coordinate in the image and is convolved with the source image S. This is done, to calculate its product or difference product sum to get the best matching. The simplest way is SAD (Sum of Absolute Differences). A pixel in the search image with coordinates (*x*_*s*_, *y*_*s*_) means that the pixel with coordinates in the source image has the intensity *I*_*s*_(*x*_*s*_, *y*_*s*_). Similarly, the coorinates (*x*_*t*_, *y*_*t*_) indicates that the corresponding coordinates in the target image have the intensity *I*_*t*_(*x*_*t*_, *y*_*t*_). Therefore, the difference between the two is 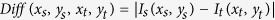.


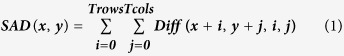



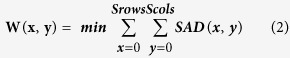


**Figure 3:**
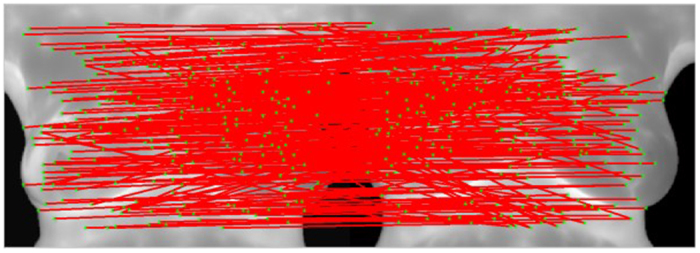
Matching with gradient direction descriptor.

However, the descriptor is a simplified description of gradient direction, and its similarity measurement merely involves the subtraction-based difference measurement among templates. This matching is defined as relaxation matching in this study. Its advantage is that most points can be matched, but its disadvantage is that there are too many mismatched points, as shown in Fig. 3. To solve the problem, this study adopted stricter Shape Context descriptor for the initial matching to obtain correct matching vectors for eliminating mismatched points.

#### Results of strict matching based on Shape Context descriptor

**Figure 4:**
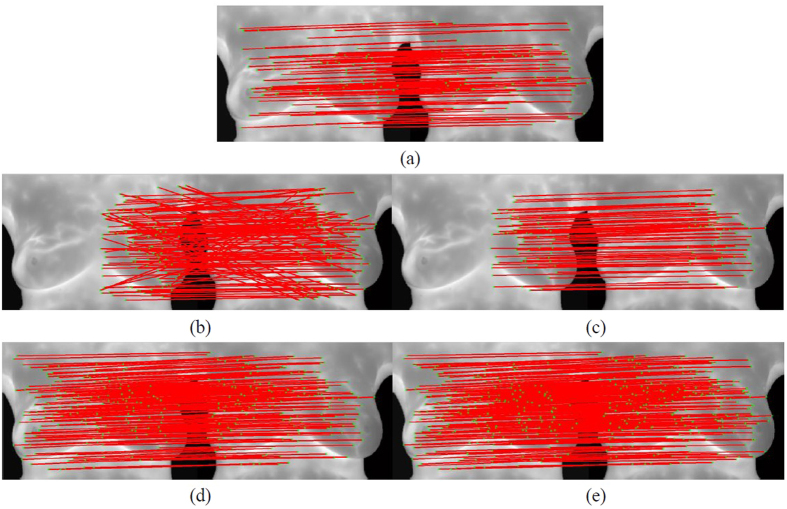
Matching with Shape Context descriptor, (**a**) Matched points obtained with Shape Context-based strict initial matching; (**b**) Non revised matched points of relaxation matching in the left area; (**c**) Modified matched points of relaxation matching in the left area; (**d**) Revised matched points of relaxation matching in two areas; (**e**) Matched points after matching vector-based revision with revised Shape Context matched points show that mismatched points can be eliminated after matched points are constrained by matching vectors.

**Figure 5:**
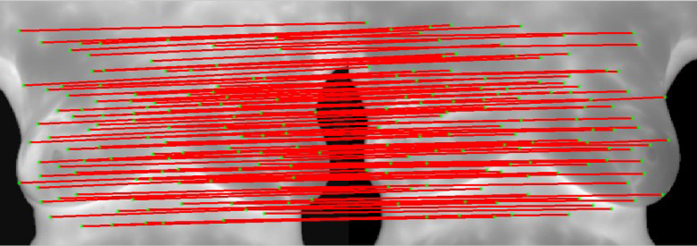
Results of matching of feature points (represented by the red lines).

**Figure 6:**
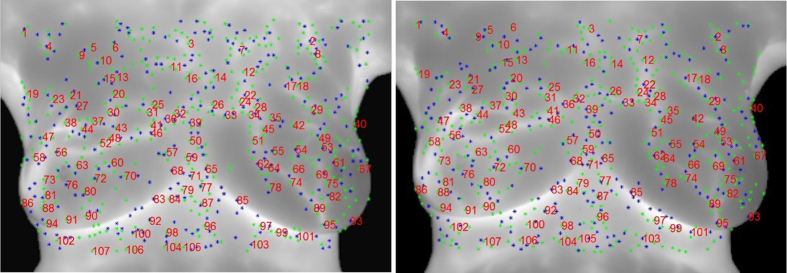
Results of matching of feature points (represented by numbers).

Figure 4 shows the modified matching of matched points. Since the degrees to which images of both breasts change are different at multiple time points, the matching vector training area in this study is divided into the left area and the right area. First, Shape Context is used for strict initial matching to obtain correct matching vectors of the two areas. As shown in Fig. 4(a), the matched points are few but stable. Therefore, the combination of the matching direction derived from the training and errors in the acceptable range could eliminate mismatched points obtained via the relaxation matching. Figure 4(b) and (c) refers to wrong points of relaxation matching in the left area. After the constriction of correct matching vectors, mismatched points could be eliminated and correct matched points in the left breast area could be obtained. Figure 4(d) indicates the modified matched points in relaxation matching. Then, matched points obtained with Shape Context in Fig. 4(a) are added to give all the correct matched points in the two images, as shown in Fig. 4(e). However, the cluster of matched points may result in low effectiveness, so adaptive non-maximal suppression method^17^ is used to avoid control point pairs clustering, as shown in Figs 5 and 6.

#### Results of competitive winner-guided optimal corresponding: adjustment of control point positions for optimal registration

**Figure 7:**
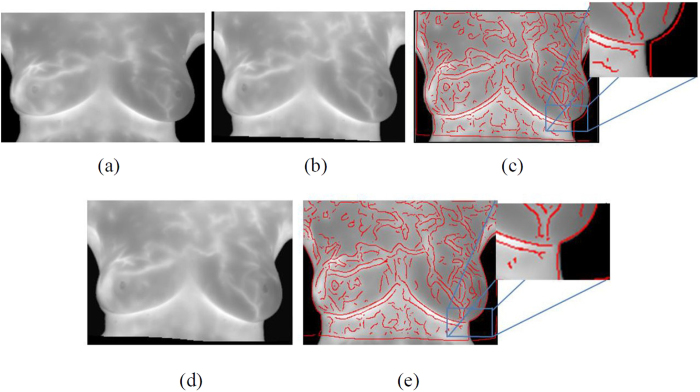
The registered results via competitive winner-guided optimal corresponding algorithm, (**a**) Target images; (**b**) The registered result of source image without competitive winning unit; (**c**) Canny boundary of (**b**) overlaying on (**a**); (**d**) the registered result of source image with competitive winning unit; (**e**) Canny boundary of (**d**) overlaying on (**a**).

**Figure 8:**
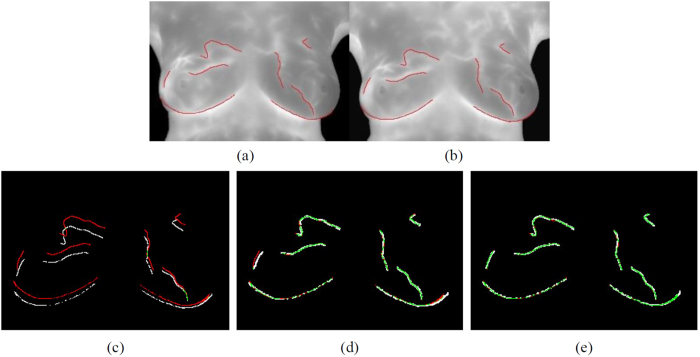
The effectiveness of the proposed algorithm, (**a**,**b**) original IR images with representative lines (red lines) at different time points. (**c**) Before registration (Coincidence rate 4.08%); (**d**) After registration by TPS transform (Coincidence rate 72.21%); (**e**) After registration by proposed algorithm (Coincidence rate 88.54%).

Because of the different basic temperatures of human bodies, the location of control points in longitudinal images may produce errors and result in poor image registration as shown in Fig. 7(c). Accordingly, this study proposes the competitive winner-guided optimal corresponding algorithm to adjust control points to correct positions. Results shown in Fig. 7(e) illustrate that the algorithm can improve the robustness and accuracy of image registration. To evaluate the effectiveness of the proposed algorithm, two radiologists with 12- and 30- year experience in breast cancer delineated the representative lines on the infrared breast images of different time points, shown as Fig. 8(a) and (b). In the Fig. 8(c), the white line refers to the source image, and the red line refers to the target image. The green line refers to the overlaid parts. Before registration, the coincidence rate is 4.08%. After registration, the source image would be deformed as the target image. In this study, the control point pairs are selected and matched automatically by the proposed automatic feature detection and matching algorithm, respectively. The control points are adjusted to the optimal positions via competitive winner-guided optimal corresponding algorithm for registration. The deformed delineated line of the source image (white) after registration is overlaid with the delineated line of the target image (red), shown as Fig. 8(d). The coincidence rate is 72.21%. Figure 8(e) shows the registered result by adjusting the control point positions via competitive winner-guided optimal corresponding algorithm, in which the coincidence rate is 88.54%.

#### Results of registration of longitudinal infrared breast images

**Figure 9:**
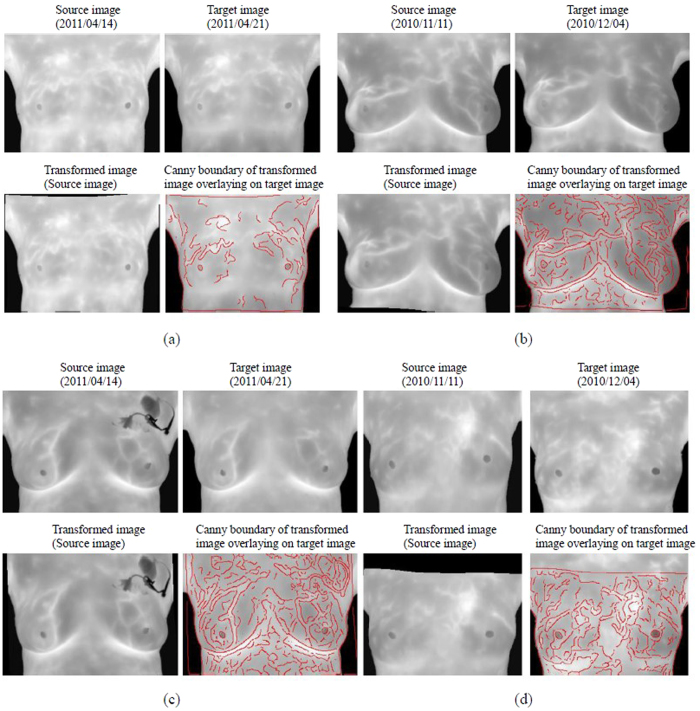
Results of infrared breast image registration, (**a**) Type1 (example: case43); (**b**) Type2 (example: case14); (**c**) Type3 (example: case21); (**d**) Type4 (example: case51).

The proposed algorithm is applied to two experiments. In the experiment 1, four subjects with different pathological features are adopted, and the details are indicated in Table 1. These subjects’ infrared images with different image characteristics are divided into four types for purposes of discussion. It demonstrates that longitudinal image registration algorithm developed in this study is robust and applicable for infrared breast images. The results are shown in Fig. 9, where source images after Canny edge algorithm are overlaid on target images for visually comparison as a direct registration evaluation. SSIM (structural similarity index) is used to play the role of an objective assessment. In the experiment 2, infrared images of ten cases are subsequently used to compare manual registration and the proposed algorithm in this study. The evaluation is based on the positions of the error of markers.

Type 1: Infrared images at different times with small deformation and slight change of heat patterns.

Case43 reveals that despite the basic body temperature being slightly alttered, the heat patterns of images are almost the same with little displacement or deformation at different time images. The registration is consequently very effective. SSIM value before registration is 0.57, and after registration is 0.60 with a 5.32% rate of increase.

Type 2: Infrared images at different times with large deformation and slight change of heat patterns.

According to the images, the position of the right breast in the target image of Case14 is higher than that in the source image. Moreover, the subject has larger breasts, which are more likely to be deformed. The proposed algorithm with fault-tolerance and error resilience for matching results in the registered results more accurately. SSIM value before registration is 0.58, and after registration is 0.81. There is a 39.59% rate of increase.

Type 3: Infrared images at different times with small deformation and big change of heat patterns.

Case21 is a difficult case for image registration. Images show that the left breast in the source image is equipped with a device which affects the feature detection and matching in the left breast. However, the registration is completed after adjusting the locations of control points via the competitive winner-guided optimal corresponding. SSIM value before registration is 0.73 and after registration is 0.79 with a rate of increase.

Type 4: Infrared images at different times with large deformation and big change of heat patterns.

Case 51 is the most difficult case for image registration. Not only does the breast have a large amount of displacement, but its heat pattern displays a considerable change. The heat patterns in the target image are brighter and have a wider distribution than the source image. Despite this, the above problem is solved in this study with relaxation matching, which can detect adequate feature point pairs. SSIM value before registration is 0.06 and after registration is 0.51 with a 74.33 % rate of increase.

### Performance analysis

**Figure 10:**
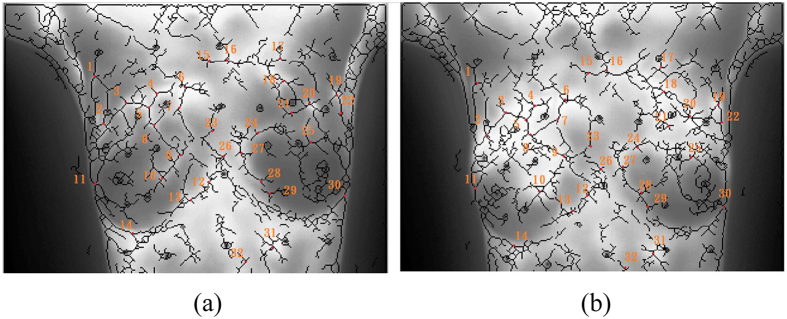
Results of manual selection of control point pairs, (**a**) Source image; (**b**) Target image (the same number represents the same control point pair; red points indicate control point positions)

**Figure 11:**
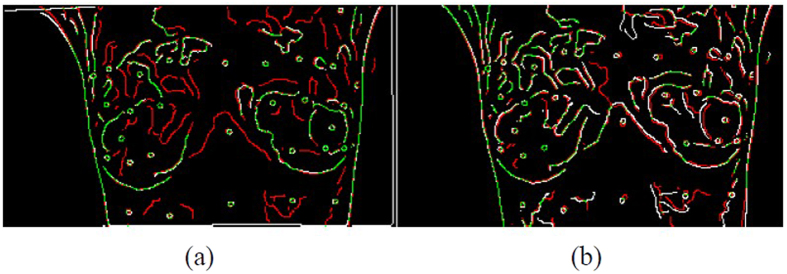
The registered results by automatically- and manually-selected control points: canny edges from the target image and the source image after registration with the same time, (**a**) The registered result of automatically-selected control points by the proposed algorithm; (**b**) The registered result of manually-selected control points. (The white line refers to the source image; the red line refers to the target influence and the green line refers to the overlaid parts).

Longitudinal infrared images of ten subjects are demonstrated in this study. The ten cases are photographed for 7 to 16 times on the same day with markers unmoved. In the process, the electric blanket simulated the change to the high-temperature tissue of patients with breast cancers so as to find out registration errors between manually-selected control points and the proposed automatic algorithm in this study. As shown in images, the experiment control points are selected for eight persons, two of whom had the experience in infrared registration while six persons didn’t. In average, about 30 control points are selected, Fig. 10 shows the positions of control point pairs of one of 10 cases. Meanwhile, the average registration time of each subject is recorded. There are about 10 time points of each subject, i.e., each subject was photographed 10 IR images, experiment results disclose that the average time for the manual registration of each subject is about 5 hours/case at each time point. Table 2 shows the difference of marker positions between the source image and the target image, in which the results were repeated two to three times to obtain the optimal registration result. However, the same cases are implement by the proposed algorithm automatically, and the details are shown as Table 3. It takes only about 0.75 hours for automatic registration, with higher registration accuracy than that of manual registration. The registered results by automatically-selected and manually-selected control points are shown in Fig. 11(a) and (b), respectively. Canny edges are performed to compare the registered results, which from the target image and the source image after registration. The white line refers to the source image, the red line refers to the target influence and the green line refers to the overlaid parts. Obviously, the registered result of automatically-selected control points by the proposed algorithm (Fig. 11(a)) has more green line, which means the performance is better than and the registered result of manually-selected control points (Fig. 11(b)).

**Figure 12:**
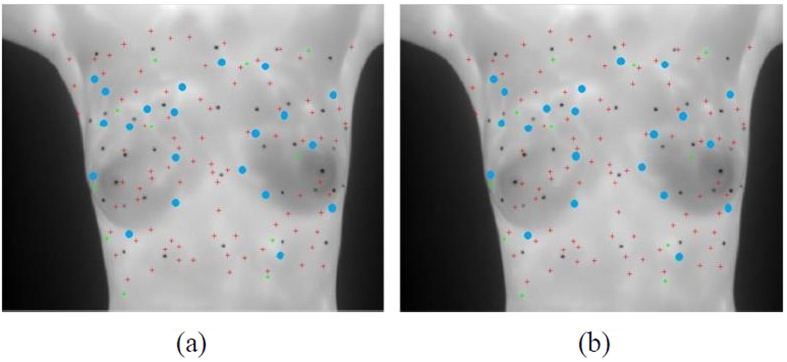
The control points are selected by automatically and manually for registration, (**a**) Source image; (**b**) Target image (the red cross points are automatically-selected control points via the algorithm; the green star points are manually-selected control points and blue circular points are repeatedly-selected control points between the two).

In addition, this study analyzes the relationships between manually- and automatically-selected control points based on the comparisons of repetition rate and distribution uniformity. Figure12 and Table 4 show that repetition rates of automatic registration and manual registration range from 55% to 85%. The difference of positions and quantity of features between two selections of control points is because of the cognitive differences in human brain, cost time, the error by manual clicking features. In addition, repetition rate varies with selector experiences and the more experience, the slightly higher repetition rate. In comprehensive consideration of average registration accuracy, cost time and quantity of control points, a higher performance algorithm in infrared image registration is developed in this study.

## Method

This study was approved by the Institutional Review Boards of the National Taiwan University Hospital (NTUH). All the experimental methods were carried out in accordance with the approved guidelines. Written informed consent was obtained from all patients involved in this study. From July 2011 to Jan 2013, 61 patients between the ages of 25 to 72 (average age of 50) recruited under an institutional review board-approved protocol, and suffered from breast cancer and were being treated with chemotherapy were examined using the Quantitative Dual-Spectrum Infrared system.

In all cases, diagnosis and chemotherapy treatment of carcinomas corresponded strictly to criteria defined by the hospital. Two IR cameras (FLIR systems) are employed to measure IR radiation in the 3–5 μm and 8–9.2 μm wavelength bands. The detectors are composed of 320*256 elements. The spatial and temperature resolutions are about 0.6 mm and 0.02 degrees Celsius. Infrared breast feature point matching and the optimal registration technology mentioned in this study introduce great effectiveness and accurate registration. Below are detailed procedures.

### Feature points detection

#### Modified Harris corner detection

Modified Harris corner detection^17^ was applied in this study. Harris corner detection considers the differential of the corner score (autocorrelation) with respect to direction. However, there are many non-local maximum feature points detected by this detector, which would result in the feature points to be clustered. Therefore, this feature detection adopts a non-maximum algorithm^17^ which overcomes the clustered feature point problem and creates representative feature points in infrared images through the selection of the adaptive suppression radius.

#### Automatic vascular intersections detection

The analysis of eigenvalues of Hessian matrix in each pixel can lead to its eigenvectors which can be used to detect the intensity change in images and estimate the geometric shape (vascular distribution) of its area. In the previous study^16^, the morphology thinning technique is applied to obtain the skeleton of the vascular map and then to select the vascular intersections manually. In this study, automatic vascular intersections detection is developed. The vascular skeleton image is subsequently taken for area labeling, which connects neighboring pixels and labels each independent area one by one. The vascular intersections could be defined. However, the detected intersections may be with a clustered problem as well. The barycenter of these intersections is then calculated and taken as the representative vascular intersections automatically.

### Feature points matching and optimization of image registration

**Figure 13:**
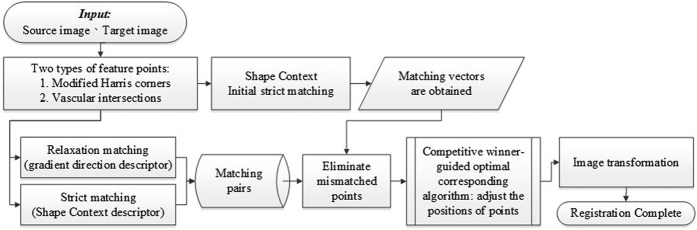
The flowchart of the proposed registration algorithm.

After feature detection, Shape Context is used to establish feature point descriptor and combines it with the gradient direction descriptor for relaxation matching to match points which could not be matched by Shape Context. The flowchart of this study is shown as Fig. 13.

#### Shape Context descriptor and matching vector

As its name suggests, Shape Context is an algorithm which divides the boundary of shape into smaller parts to record the number of contour points and establish a shape descriptor for matching. The method hypothesizes the shape of objects and describes objects with limited point sets (N) in objects or on their external contours. In this study, feature point distribution of an infrared image is taken as a contour. To describe the contour of these point sets, each point is regarded as a center to build a coordinate system. The information around the point is then recorded in each bin of the contour histogram. The distribution of the remaining N-1 contour points in the coordinate is calculated and taken as the Shape Context of the contour point. Finally, the similarity of the Shape Context descriptors is calculated by considering two points *p* and q that have normalized K-bin histograms *g*(k) and h(k), and the cost function C_s_ is used to evaluate the cost of matching the two points. The equation is


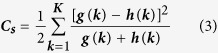


After Shape Context similarities of all points are obtained, the cost function matrix can be established. Then, each point *p*_*i*_ on the shape and *q*_*j*_ on another shape would be one-to-one matched by minimizing the cost of matching *H*(π) by the following equation:


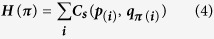


**Figure 14:**
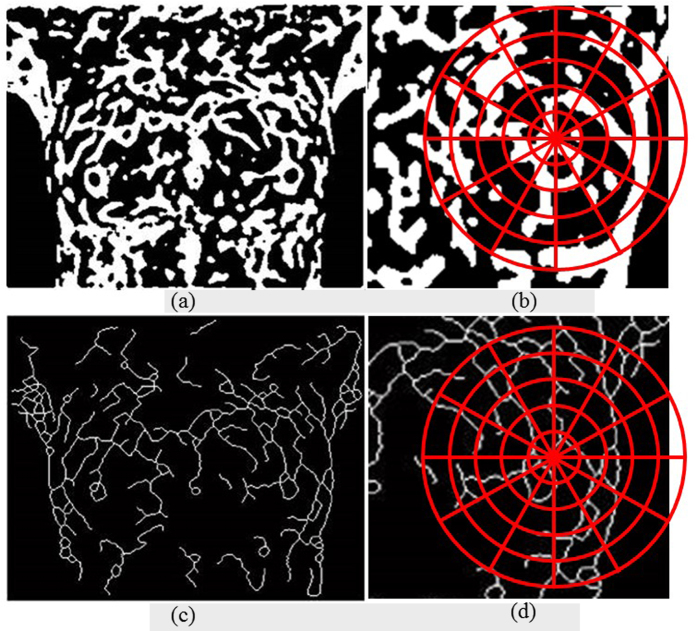
The diagram of establishment of Shape Context descriptor.

Because of heat patterns and the vascular skeleton map of IR breast images taken at different time points are similar with slight rotation of human body, Shape Context is used to establish the descriptor of feature points. In this study, the coordinate system is set to 60 regions with 12 angles at the center of the feature points and 5 segmented-regions at the outside of each angle direction. Beside the N-1 contour information is adopted, the vascular skeleton map (Fig. 14(a)) and the binary image (Fig. 14(c)) are also applied to establish the Shape Context descriptor (Fig. 14(b) and (d)), respectively, shown in Fig. 14. It means that each feature point has a 120 regions of the Shape Context descriptor.

After the descriptor establishment, the proximity matrix is used for the optimal matching of feature points. Assume the position of the optimal solution in the proximity matrix is (*i, j*) and the cost is *C*_*s*(*i, j*)_, the sub-optimal solution of the *i* direction is *C*_*s*(*isec, j*)_ and the sub-optimal solution in the j direction is *C*_*s*(*i, j*sec**)_. In addition, points can be selected as matched points must also satisfy the following conditions:


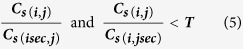


Where *T* is (Shape Context radius - feature point suppression radius) / (Shape Context radius).

#### Image transformation: TPS algorithm

After feature point matching, TPS transformation is used to transform images for registration. The equation is as following,


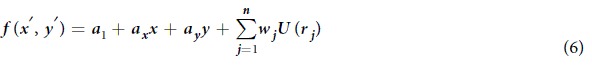


where n is the number of control points; *j* is *j*th control point; (*x, y*) refers to the coordinate in the original image; (*x*′, *y*′) is the new coordinate derived after transformation; *a*_1_, *a*_*x*_ and *a*_*y*_ are linear parameters and *w*_*j*_ indicates n×1 non-affine coefficients.


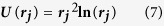


It mainly describes the influence of forces on different control points. TPS generates deformation according to the deformation model. *r*_*j*_ that refers to the distance between (*x, y*) and *j*th coordinate point,


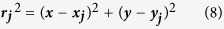


To solve the affine coefficient, it is necessary to obtain *a*_*1*_, *a*_*x*_, *a*_*y*_ and *w*.

Make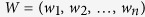, 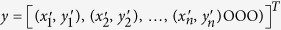 and define the following matrix:


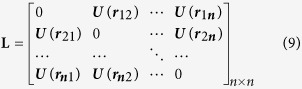


Where *r*_*ij*_ refers to the distance between *i*th and *j*th control points in the original image.


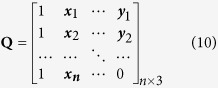



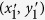
 is the coordinate of the control point in the original image. After all parameters are obtained from the matrix equation *L*^−1^*Y* = *W*. The Eq. 6 is used to obtain the coordinate of the target point position after transformation and further to accomplish the preliminary image registration.

#### Image registration optimization: competitive winner-guided optimal corresponding

Although the heat pattern of body surface is like as fingerprint, infrared breast images taken at different time points would result in slight change to the heat pattern due to different physiological or environmental conditions. It causes the matched feature points at a slightly different position. Therefore, it is necessary to make a further adjustment of control point positions to achieve the optimal image registration.

**Figure 15:**
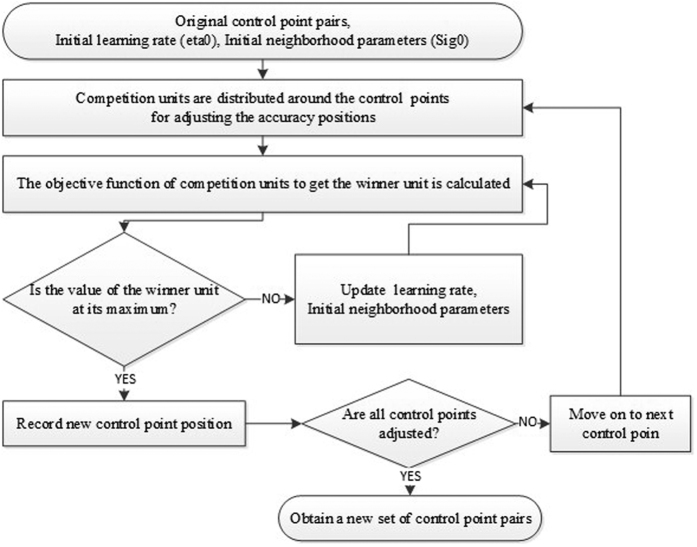
The flowchart of competitive winner-guided optimal corresponding algorithm.

Self-organizing map (SOM) is adopted in this study to search optimal corresponding points, and the flowchart is shown in Fig. 15. The main concept is to adjust positions of control points for optimal registration by self-organizing competition. SOM was proposed by Kohenen in 1980^42^. In artificial neural network, it is unsupervised learning. It has been widely explored in previous studies and applied to various issues. An SOM has a simple topological node set (e.g., two-dimension network) and a node in the distance function. The node is repeatedly mapped to a k-dimension space (conditional space). Specifically, a node N in *i*th iteration is referred as *f*_*i*_(*N*), while the initial mapping *f*_0_ is random. In the follow-up iteration, a data point P is chosen (winning condition), and the closest mapping point is *N*_*p*_ (winning unit). The remaining nodes are adjusted for mapping according to their distance from *N*_*p*_. In this way, each node would move from the initial state to a (neighboring) point with the similar condition in the k-dimension space. The equation is


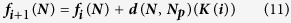


The equation of the distance function *d*(*N, N*_*p*_) with (x, y) as the coordinate is


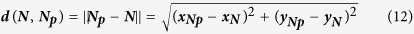


*K*(*i*) is the kernel function which includes learning rate function *EtaN* and neighboring function *Si*g*N*. It denotes the iterative adjustment of the connection weight. The equation is


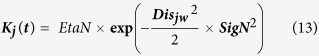


where


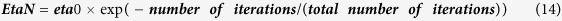



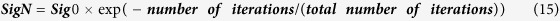


The learning rate function is used to adjust parameters of the competitive force and the iteration time. *eta*0 is an initial learning rate. The neighboring function restricts the adjustment range of the competition unit according to the relationship between the number of iterations and the total number of iterations. *Sig*0 is the initial neighborhood parameter.

In this study, competition points, also called competition units, are distributed around feature points which need to be adjusted and these competition units are taken as control points. After registration, the competition unit with maximum mutual information is set as the winning unit. Then, competitive winner-guided optimal corresponding points are adjusted until convergence according to SOM learning rules. The convergence condition is that adjusting the position of the winner unit, and which remains the same after three iterations. Finally, the optimal positions of control points could be found to achieve the optimal longitudinal image registration. The limitation of the proposed algorithm is that the subject undergoes a mastectomy to reduce the chances of getting breast cancer.

## Conclusion

In recent years, many studies on the registration of infrared breast images have been laid upon sequential dynamic infrared imaging^43^,^44^. Longitudinal IR image registration is an essential step toward the quantitative pixel-wise analysis of the heat energy and pattern change in a time course study. However, it the problem is a difficult one for a variety of reasons: individual differences in subjects, the inability for the photographed pose to be fixed at different time, and the impracticality of keeping markers attached on a body surface for weeks. Selecting feature points and matching are often accomplished manually for longitudinal IR image registration but the process is time consuming and laborious^16^. This study overcomes the problems and develops a new automatic marker-free longitudinal IR image registration algorithm based on detecting two representative fiducial feature points automatically. The gradient direction of images and Shape Context are then used to establish a descriptor for two types of feature points. The matching vector obtained from the sharp context method is used to limit and eliminate the mismatching points, which is matched by the relaxation matching, to obtain the most optimal feature point matching. The competitive winner-guide optimal corresponding is employed to adjust the accurate positions of control points, which are obtained by matching. Finally, TPS transformation is used to align the source image and the target image. Results of two aspects tell that the effectiveness of automatic registration is better than manual registration with the mean error being 0.91 pixels, and 85% faster than manual registration. It demonstrates that the proposed automatic marker-free longitudinal infrared image registration improves the registration efficiency, and may serve as a tool for longitudinal registration to help the assessment of the chemotherapy response and breast cancer detection on IR images. The vision of this study is that females can perform frequent or regular self check-up with a low-cost, high-performance and harmless breast imaging system to achieve early detection of breast cancer or effective monitoring of treatment response in the future.

## Additional Information

**How to cite this article**: Lee, C.-Y. *et al*. Automatic Marker-free Longitudinal Infrared Image Registration by Shape Context Based Matching and Competitive Winner-guided Optimal Corresponding. *Sci. Rep.* **7**, 39834; doi: 10.1038/srep39834 (2017).

**Publisher's note:** Springer Nature remains neutral with regard to jurisdictional claims in published maps and institutional affiliations.

## Figures and Tables

**Table 1:** The pathological features of four cases.

Case Number	Clinical Stage	Age/Gender	Method	Tumor site description (tumor site)	size(cm)
C14	Stage IIIB	58/Female	SONO	9 o’clock position on the right breast, 4 cm from nipple. (R’t 9/4)	2.8*2.3
			MRI	Outer quadrant of the right breast. (R’t outer half)	4.5
C21	Stage IIB	46/Female	SONO	10 o’clock position on the right breast, 6 cm from nipple. (R’t 10/6)	1.5*1.2
			MRI	Upper outer quadrant and upper hemisphere of the right breast. (R’t UOQ and upper half)	8
C43	Stage IIA	47/Female	SONO	2 o’clock position on the right breast, 6 cm from nipple. (R’t 2/6)	3.37*2.53
			MRI	Upper inner quadrant of the right breast. (R’t UIQ)	3.9
C51	Stage IIB	54/Female	SONO	11 o’clock position on the right breast, 5 cm from nipple. (L’t 11/5)	2.93*1.93
			MRI	Upper inner quadrant of the left breast. (L’t UIQ)	3

*UOQ: Upper Outer Quadrant; UIQ: Upper Inner Quadrant.

**Table 2:** Results of registration manually (The average registration time (about 10 time points) of each subject was 5 hours/case).

subject No.	The difference of marker positions between the source- and the target image (unit: pixel)		
	min	max	mean
subject 1	0.0265	7.0115	1.8081
subject 2	0.1564	5.4312	1.9761
subject 3	0.9223	7.6552	1.2768
subject 4	0.0763	5.6254	1.2266
subject 5	0.6623	4.3612	1.9579
subject 6	0.2638	8.6323	2.8513
subject 7	0.1085	6.851	3.9579
subject 8	0.4025	6.8521	2.9479
subject 9	0.4955	9.4811	2.9826
subject 10	0.9925	6.2212	2.0965

**Table 3:** Results of registration automatically by the proposed algorithm (the average time for the registration (about 10 time points) of each subject was 45 mins/case).

subject No.	The difference of marker positions between the source- and the target image (unit: pixel)		
	min	max	mean
subject 1	0	2.01	0.8081
subject 2	0	2.23	1.0761
subject 3	0	2.01	0.8768
subject 4	0	2.57	0.8266
subject 5	0	3.85	0.9579
subject 6	0	2.59	0.8513
subject 7	0	3.85	0.9595
subject 8	0	3.40	0.9479
subject 9	0	2.48	0.8826
subject 10	0	3.21	0.8652

**Table 4:** Relationship between manually-and automatically-selected control points.

		Repetition Relationship and Distribution Relationship with Automatic Selection			
Manual 1–6 for Those Without Experience; Manual7–8 for Those With Experience		Average Number of Manual Selection	Average Number of Repetition	Average Rate of Repetition	Comparison of Evenness
Those Without Experience	Manual 1	32.12	20.73	64.54%	Poor
	Manual 2	31.85	18.38	57.71%	Poor
	Manual 3	32.56	17.82	54.73%	Poor
	Manual 4	31.95	16.96	53.08%	Poor
	Manual 5	29.61	17.23	58.19%	Poor
	Manual 6	30.42	18.99	62.43%	Poor
Those with Experience	Manual 7	32.12	25.89	83.19%	Poor
	Manual 8	31.85	22.95	71.74%	Poor
